# Data of innovation ambidexterity as a mediator in the absorptive capacity effect on sustainable competitive advantage

**DOI:** 10.1016/j.dib.2020.105200

**Published:** 2020-01-29

**Authors:** Astadi Pangarso, Endang Siti Astuti, Kusdi Raharjo, Tri Wulida Afrianty

**Affiliations:** a Business Administration Department, Administrative Science Faculty, Brawijaya University, Indonesia; b Business Administration Department, Telkom University, Indonesia

**Keywords:** Innovation ambidexterity, Absorptive capacity, Sustainable competitive advantage, Indonesian higher education

## Abstract

This data article shows the nexus between absorptive capacity (X), innovation ambidexterity (Y1) and sustainable competitive advantage (Y2). There are three nexus points between the constructs, namely the direct nexuses of X to Y1, X to Y2 and the indirect nexus from X to Y2 through Y1. The raw data of 530 self-administrated questionnaires were obtained from 64 non-vocational private higher education institutions in the Bandung area of West Java, Indonesia. Data analyzing were conducted using SPPS and Smart PLS. The data are useful as the data can be reproduced, reused and reanalysed. This data article also opens up better research opportunities going forward through collaboration with other researchers.

**Table:** Specifications Table

Subject	Business and International Management
Specific subject area	Absorptive Capacity, Innovation Ambidexterity, Sustainable Competitive Advantage
Type of data	Table Figure
How data were acquired	The data were collected using a survey with questionnaires. The data were analyzed using SPSS and Smart PLS. The link of the questionnaire: https://data.mendeley.com/datasets/z2y8gmxtrb/3#file-e6fb3164-e259-47d1-b4fc-0d0d2c41b2fd
Data format	Raw Smart PLS data
Parameters for data collection	The sample consisted of 530 respondents. The data were collected using a self-administrated questionnaire from 64 non-vocational private higher education institutions.
Description of data collection	The questionnaire data were collected through a survey. The collection of questionnaires for each non-vocational private higher education institutions was done through 1 key person/enumerator. The researcher submits the research permission application letter to the non-vocational private higher education institutions with the help of the enumerators. After being allowed to distribute the questionnaire, the researcher discusses with the enumerators how the technical implementation of the questionnaire is distributed. Researcher offer two types of questionnaires to enumerators. The questionnaire can be distributed offline and online following the policies of each non-vocational private higher education institutions. The researcher entrusted the questionnaire in the form of a hardcopy or a link of a google form to the enumerators to be distributed to respondents. The researcher communicate with the enumerators by the WhatsApp number or cellular phone so the process of collecting questionnaire data could be monitored and quick collected from each non-vocational private higher education institutions.
Data source location	Institution: Non-vocational private higher education institution City/Town/Region: Bandung area, West Java Country: Indonesia
Data accessibility	Repository name: Mendeley Data Data identification number: 10.17632/z2y8gmxtrb.3 Direct URL to data: https://data.mendeley.com/datasets/z2y8gmxtrb/3

**Table:** 

**Value of the Data** • This data article has the potential for the research community to replicate it using different quantitative data software processing. This is in order to be able to compare the results between the software (for example: AMOS, LISREL, Warp PLS, PLS using R, Adenco etc). • This data article is expected to open up opportunities for collaboration with other researchers related to future research with the following constructs: absorptive capacity, innovation ambidexterity and sustainable competitive advantage. • The Indonesian leaders of the non-vocational private higher education institutions, the LLDIKTI (Indonesian higher education administrator institution) and also the researchers, will get benefits from this data related to increasing the non-vocational private higher education institutions sustainable competitive advantage. • This data article is useful because it will become the basis for the interpretation of the next research article publication related to the mediation role of innovation ambidexterity on the effect and prediction of absorptive capacity to sustainable competitive advantage.

## 1 Data description

The questionnaire data consisted of 3 research variables, namely absorptive capacity (AC) as the independent variable (X), innovation ambidexterity (IA) as the first dependent variable (Y1) and sustainable competitive advantage (SCA) as the second dependent variable (Y2). The questionnaire consists of 60 statement indicators that must be answered based on the Likert scale of 1–5 (very disagree to very agree). Variable X consists of 19 indicator items adopted from Ref. [1]; variable Y1 consists of 9 indicator items adopted from Refs. [2,3]; and variable Y2 consists of 32 indicator items adopted from Refs. [4,5]. Questionnaire data were obtained from Research Data [6].

This questionnaire belongs to the category of self-administration and therefore it needs to be tested for common method variance [7]. Self-administrated questionnaires can potentially lead to a common method bias. Therefore this questionnaire needs to be checked in order to whether this research is free from common method bias. The evaluation of common method variance (CMV) using the Harman single factor test has been carried out and the variance value is 38.837%. If the percentage variance is below 50%, then it can be said that the measurement of the research indicators has passed the common method bias. Table 1 states the results of the Harman single factor test for CMV testing using SPSS.

**Table 1:** Harman single factor test Total Variance Explained.

Component	Initial Eigenvalues			Extraction Sums of Squared Loadings		
	Total	% of Variance	Cumulative %	Total	% of Variance	Cumulative %
1	23.302	38.837	38.837	23.302	38.837	38.837
2	3.004	5.007	43.844			
3	2.635	4.392	48.237			
4	1.991	3.318	51.555			
5	1.564	2.607	54.161			
6	1.471	2.452	56.614			
7	1.446	2.409	59.023			
8	1.204	2.007	61.030			
9	1.165	1.942	62.972			
10	1.060	1.766	64.738			
11	.987	1.645	66.383			
12	.869	1.448	67.830			
13	.821	1.369	69.199			
14	.781	1.302	70.501			
15	.744	1.240	71.741			
16	.724	1.207	72.948			
17	.686	1.144	74.092			
18	.677	1.128	75.220			
19	.655	1.092	76.312			
20	.617	1.028	77.340			
21	.608	1.013	78.352			
22	.589	.982	79.334			
23	.570	.951	80.285			
24	.551	.918	81.203			
25	.545	.908	82.111			
26	.494	.823	82.934			
27	.475	.791	83.725			
28	.465	.776	84.501			
29	.458	.764	85.265			
30	.451	.752	86.016			
31	.437	.728	86.745			
32	.430	.716	87.461			
33	.400	.666	88.127			
34	.396	.660	88.787			
35	.383	.638	89.425			
36	.361	.602	90.026			
37	.351	.584	90.611			
38	.341	.568	91.178			
39	.332	.553	91.731			
40	.328	.547	92.278			
41	.315	.524	92.802			
42	.309	.515	93.318			
43	.306	.510	93.828			
44	.292	.487	94.315			
45	.292	.487	94.802			
46	.287	.478	95.280			
47	.264	.440	95.720			
48	.253	.422	96.142			
49	.243	.405	96.547			
50	.240	.400	96.947			
51	.227	.379	97.325			
52	.213	.355	97.681			
53	.210	.350	98.031			
54	.197	.328	98.360			
55	.189	.315	98.674			
56	.182	.303	98.977			
57	.173	.288	99.265			
58	.159	.264	99.529			
59	.154	.257	99.787			
60	.128	.213	100.000			

Extraction Method: Principal Component Analysis.

From the results of the descriptive statistics as can be seen in Table 2, the demographics of the respondents in this research were balanced between men and women. The highest number of educated level was a Master's. Furthermore, the research respondents were dominated by full-time lecturers.

**Table 2:** Respondent profile.

Characteristics	Sub characteristics	Frequency	Percentage (%)
Gender	Male	277	52
	Female	253	48
Education level	Bachelor	35	7
	Master	380	72
	Ph.D/DR.	115	21
Structural position	No	134	25
	Yes	396	75
Structural Position Name	Lecturer	277	52
	Quality Assurance	52	10
	Leader	201	38

**Fig. 1:**
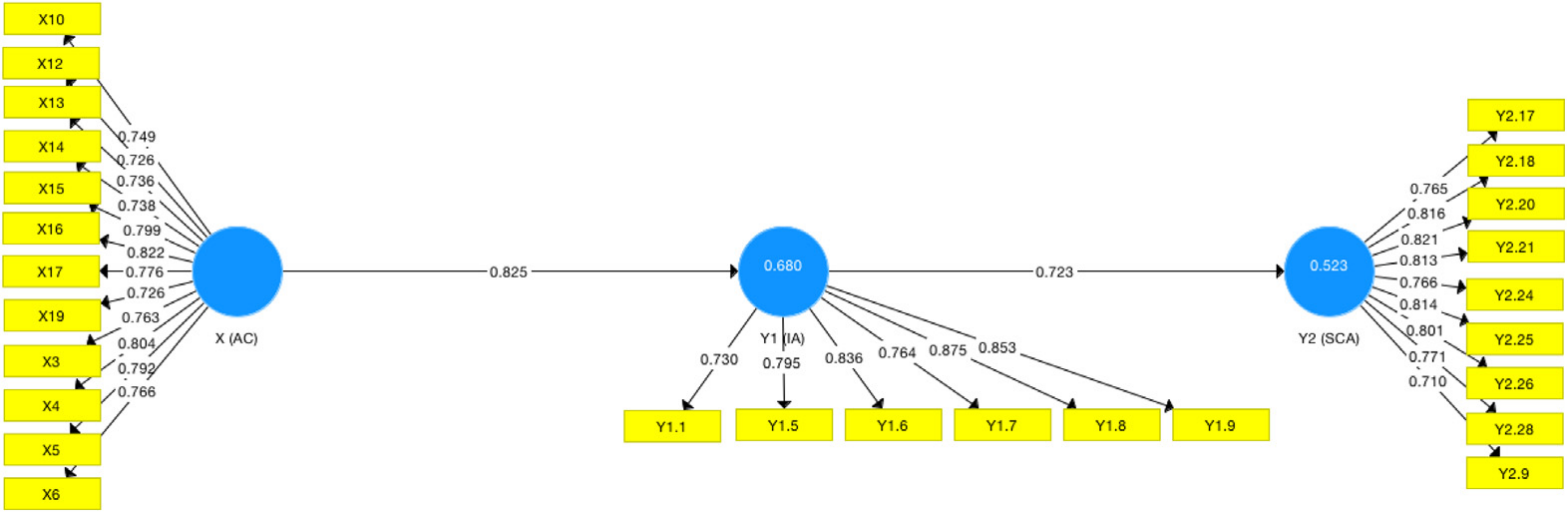
Measurement model evaluation.

The questionnaire data analyzing were done using the smart PLS protocol according to Ref. [8]. Data analyzing using smart PLS consists of the measurement model evaluation and structural model evaluation. The measurement model calculation can be seen sequentially in Table 3, Table 4, Table 5. Smart PLS preparation begins from assessing the measurement model through indicator reliability, internal consistency reliability, convergent validity and discriminant validity [8]. The reliability of the indicator is known by the loading factor value (>0.708), which means that the indicator is reliable. The factor loading in Table 3 for each indicator must be more than 0.708. If the factor loading value is less than 0.708, then it will be removed and not included in the next evaluation process. Only the indicators with loading factor values of 0.708 or more are included in the next evaluation process. From Fig. 1, it can be seen that there are indicators whose values are the same or more than 0.708.

**Table 3:** Loading factors.

	Absorptive Capacity	Innovation Ambidexterity	Sustainable Competitive Advantage
X10	0.754		
X11	0.690		
X12	0.718		
X13	0.724		
X14	0.723		
X15	0.775		
X16	0.804		
X17	0.765		
X18	0.677		
X19	0.710		
X2	0.696		
X3	0.753		
X4	0.799		
X5	0.780		
X6	0.773		
X7	0.706		
X8	0.610		
X9	0.603		
Y1.1		0.755	
Y1.2		0.714	
Y1.3		0.645	
Y1.4		0.722	
Y1.5		0.801	
Y1.6		0.781	
Y1.7		0.713	
Y1.8		0.825	
Y1.9		0.816	
Y2.1			0.525
Y2.10			0.560
Y2.11			0.634
Y2.12			0.529
Y2.13			0.553
Y2.14			0.582
Y2.15			0.551
Y2.16			0.531
Y2.17			0.705
Y2.18			0.767
Y2.19			0.484
Y2.2			0.474
Y2.20			0.761
Y2.21			0.759
Y2.22			0.683
Y2.23			0.669
Y2.24			0.760
Y2.25			0.798
Y2.26			0.777
Y2.27			0.612
Y2.28			0.733
Y2.29			0.665
Y2.3			0.619
Y2.30			0.012
Y2.31			0.055
Y2.32			0.046
Y2.4			0.589
Y2.5			0.695
Y2.6			0.676
Y2.7			0.406
Y2.8			0.661
Y2.9			0.704
X1	0.658

**Table 4:** CR and AVE values.

	Composite Reliability (CR)	Average Variance Extracted (AVE)
Absorptive Capacity	0.945	0.588
Innovation Ambidexterity	0.920	0.657
Sustainable Competitive Advantage	0.936	0.620

**Table 5:** HTMT values.

	Absorptive Capacity	Innovation Ambidexterity	Sustainable Competitive Advantage
Absorptive Capacity			
Innovation Ambidexterity	0.897		
Sustainable Competitive Advantage	0.831	0.789

Internal consistency reliability is measured based on composite reliability values (CR) > 0.70, which means that the research variable is reliable. The convergent validity is represented by the value of Average Variance Extracted/AVE (>0.50), which means that the variable can explain more than 50% of the variance of the indicators. The AVE value in Table 4 for each variable must be higher than 0.50.

Furthermore, the discriminant validity uses HeteroTraitMonoTrait (HTMT) values. The HTMT or discriminant validity values in Table 5 for each research variable must be less than 0.90. The HTMT values of the research variables were below 0.90 [9], which means that the research variables have good discriminant validity.

All of the indicators and variables have passed the measurement model evaluation process and have fulfilled all of the rules of thumb, as can be seen in Fig. 1.

**Fig. 2:**
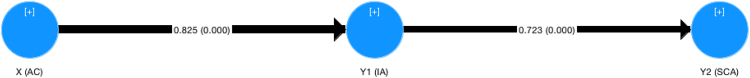
Structural model evaluation.

After evaluating the measurement model, it is followed by an evaluation of the structural model consisting the values of inner VIF, path coefficients, specific indirect effect, R^2^ and Q^2^ [8]. The Fig. 2 and Table 6 show the structural evaluation model in sequence from Table 6 through to 10. The inner VIF structural model for all of the research variables in Table 6 has fulfilled the cut-off in the range of 0.20 up to less than 5, which means that all of the research variables are free from collinearity problems.

**Table 6:** Inner VIF values.

	Absorptive Capacity	Innovation Ambidexterity	Sustainable Competitive Advantage
Absorptive Capacity		1.000	
Innovation Ambidexterity			1.000
Sustainable Competitive Advantage			

The number of hypotheses in the structural model consists of 2 direct nexus and one indirect nexus. The direct nexuses are X to Y1 and Y1 to Y2. The indirect nexus is X to Y2 through Y1. Table 7 shows that all of the direct nexus are significant.

**Table 7:** Path coefficients.

	Original Sample (O)	Sample Mean (M)	Standard Deviation (STDEV)	T Statistics (|O/STDEV|)	P Values
Absorptive Capacity - > Innovation Ambidexterity	0.825	0.825	0.018	45.811	**0.000**
Innovation Ambidexterity - > Sustainable Competitive Advantage	0.723	0.724	0.030	23.956	**0.000**

The definition of significance of bold is if the p-value less than 0.05.

In Table 7, the rule of thumb of the direct effect between the variables shows that the p-value is smaller than 0.05 and the t-statistics value is higher than 1.96 (using a 5% confidence level).

In Table 8, the rule of thumb for the specific indirect effect between the variables shows that the p-value is less than 0.05 and the t-statistics value is higher than 1.96 (using a 5% confidence level). Table 8 shows that the indirect nexus is significant.

**Table 8:** Specific indirect effect.

	Original Sample (O)	Sample Mean (M)	Standard Deviation (STDEV)	T Statistics (|O/STDEV|)	P Values
Absorptive Capacity - > Innovation Ambidexterity - > Sustainable Competitive Advantage	0.596	0.598	0.034	17.421	**0.000**

The definition of significance of bold is if the p-value less than 0.05.

In Table 9, the rule of thumb shows that the original sample (O) value of R^2^ and the p-value are both smaller than 0.05. The original sample (O) values are higher than 0.25. Furthermore, the R^2^ values between 0.5 and 0.75 indicate that the structural model has moderate explanatory power (see Table 9).

**Table 9:** R^2^ values.

	Original Sample (O)	Sample Mean (M)	Standard Deviation (STDEV)	T Statistics (|O/STDEV|)	P Values
Innovation Ambidexterity	0.680	0.681	0.030	22.929	**0.000**
Sustainable Competitive Advantage	0.523	0.526	0.044	12.018	**0.000**

The definition of significance of bold is if the p-value less than 0.05.

In Table 10, the rule of thumb shows that the values of Q^2^ are higher than zero. All of the Q^2^ values are in the range of 0.25–0.5, which means that the structural model has medium predictive relevance.

**Table 10:** Q^2^ values.

	SSO	SSE	Q^2^ (=1-SSE/SSO)
Absorptive Capacity	6,360.000	6,360.000	
Innovation Ambidexterity	3,180.000	1,863.092	0.414
Sustainable Competitive Advantage	4,770.000	3,343.784	0.299

All of the variables have passed the structural model evaluation process and they have fulfilled all of the rules of thumb. The structural model evaluation can be seen in Fig. 2 below.

## 2 Experimental design, materials, and methods

This data article used a quantitative research method approach. The data analysis unit were organisations. The research population consisted of all non-vocational private higher education institution in the area of Bandung, West Java, Indonesia taken from Ref. [10]. The number of samples of this research were the same as the total non-vocational private higher education institutions in the Bandung area, which were 81. The sampling technique used was non-probability sampling, with saturated sampling making all of the members of the population the sample [11]. Each non-vocational private higher education institution had an average of 10 respondents, so the total number of respondents who would filled the questionnaire were 810. The questionnaire data were collected between May 2019 and September 2019. The questionnaire data that were collected and found to be suitable for the analyzing were 530 questionnaires from 64 non-vocational private higher education institutions. The response rate of the data collection was 65.43%. The data collected has fulfilled the minimum requirements of the Smart PLS sample size recommendation, with a range of 8–90 organisations for theoretical models with a significance level of 5% [8]. The data collected were analyzed into SPSS for common method variance in order to evaluate whether the research indicators are free of bias [7]. Descriptive statistics were used to know the respondent's profile.

Smart PLS was used with the considerations as follow [8]:

1. Aims to identify the key driver of a variable (measurement model)
2. Can be used to structure complex theoretical models (consisting of many indicators)
3. Can be used for small sample sizes and for data that is not normally distributed
4. Aim at analysing the latent variables (structural model)

SmartPLS was used for the measurement model evaluation and structural model evaluation [8]. The measurement model evaluation was first used in the analyzing of the Smart PLS data in order to examine the feasibility of the research indicators. All of the indicators are stated to have met the rule of thumb. The measurement model evaluation was followed by the structural model evaluation. The structural model evaluation was used to examine the nexus between the research variables with conclusions that were either significant or not. The data analyzing in the structural model evaluation used the complete bootstrapping 5000 sample method inclusive of the two-tailed BCa confidence interval method and a 0.05 confidence level.
